# Stroke distribution patterns and characteristics in Kenya’s leading public health tertiary institutions: Kenyatta National Hospital and Moi Teaching and Referral Hospital

**DOI:** 10.5830/CVJA-2017-046

**Published:** 2018

**Authors:** Kaduka Lydia, Muniu Erastus, Korir Anne, Mbui Jane, Owuor Oduor Chrispine, Kwasa Judith, Wabwire Sylvanos, Gakunga Robai, Okerosi Nathan, Opanga Yvonne, Kisiang’ani Isaac, Rotich Chepkurui Mercy, C Remick Scot

**Affiliations:** Centre for Public Health Research, Kenya MedicalResearch Institute, Nairobi, Kenya; Centre for Public Health Research, Kenya MedicalResearch Institute, Nairobi, Kenya; Centre for Clinical Research, Kenya Medical Research Institute, Nairobi, Kenya; Centre for Clinical Research, Kenya Medical Research Institute, Nairobi, Kenya; Department of Medicine, School of Medicine, College of Health Sciences, Moi University, Eldoret, Kenya; Department of Clinical Medicine and Therapeutics, Kenyatta National Hospital, Nairobi, Kenya; Kenyatta National Hospital, Nairobi, Kenya; Kenya Cancer Association, Nairobi, Kenya; Kenya Cancer Association, Nairobi, Kenya; School of Public Health, Moi University, Eldoret, Kenya; School of Public Health, Jomo Kenyatta University ofAgriculture and Technology, Nairobi, Kenya; School of Public Health, Jomo Kenyatta University ofAgriculture and Technology, Nairobi, Kenya; Maine Medical Center Research Institute, Portland, ME, USA

**Keywords:** stroke, Kenya, sub-Saharan Africa, mortality, risk factors

## Abstract

**Background:**

Cardiovascular diseases are the second leading cause of morbidity and mortality in Kenya. However, there is limited clinico-epidemiological data on stroke to inform decision making. This study sought to establish stroke distribution patterns and characteristics in patients seeking care at Kenyatta National Hospital (KNH) and Moi Teaching and Referral Hospital (MTRH), with the ultimate aim of establishing the first national stroke registry in Kenya.

**Methods:**

This was a prospective multicentre cohort study among stroke patients. The study used a modified World Health Organisation STEP-wise approach to stroke surveillance tool in collecting data on incidence, major risk factors and mortality rate. The Cochran’s Mantel–Haenszel chisquared test of conditional independence was used with p-value set at 0.05.

**Results:**

A total of 691 patients with confirmed stroke were recruited [KNH 406 (males: 40.9%; females: 59.1%); MTRH 285 (males: 44.6%; females: 55.4%)] and followed over a 12-month period. Overall, ischaemic stroke accounted for 55.6% of the stroke cases, with women being the most affected (57.5%). Mortality rate at day 10 was 18.0% at KNH and 15.5% at MTRH, and higher in the haemorrhagic cases (20.3%). The most common vascular risk factors were hypertension at 77.3% (males: 75.7%; females: 78.5%), smoking at 16.1% (males: 26.6%; females: 8.3%) and diabetes at 14.9% (males: 15.7%; females: 14.4%). Ischaemic stroke was conditionally independent of gender after adjusting for age.

**Conclusions:**

To our knowledge this is the first pilot demonstration establishing a stroke registry in sub-Saharan Africa and clearly establishes feasibility for this approach. It also has utility to both inform and potentially guide public policy and public health measures on stroke in Kenya. Important and unexpected observations included the preponderance of women affected by cerebrovascular disease and that cigarette smoking was the second most common risk factor. The latter, over time, will further impact on the clinico-epidemiological profile of cerebrovascular disease in Kenya.

African countries are undergoing an epidemiological transition characterised by socio-demographic and lifestyle changes, resulting in increased risk factors and burden of cerebrovascular disease (hereafter referred to as stroke). According to the World Health Organisation (WHO), low- and middle-income countries bear the heaviest (86%) global stroke burden,^1^ with 8% of all first-ever strokes occurring in Africa and 5% of the 30 million stroke survivors worldwide living in Africa.^2^

There are limited clinico-epidemiological data on stroke from sub-Saharan Africa to effectively inform policy and guide intervention efforts. Cardiovascular disease (CVD) is the second leading cause of morbidity and mortality in Kenya. The Kenya Health Sector Strategic Plan (KHSSP) estimates mortality rate due to CVD at 6.1%, while the WHO estimates it at 8%. Autopsy studies suggest that more than 13% of cause-specific deaths among adults could be due to CVD.^2^-^4^

Implementation of surveillance systems is necessary to monitor trends and inform prevention and management programmes.^5^,^6^ Although the WHO recommends establishment of an in-country system of surveillance and monitoring of non-communicable diseases, these systems have not been well executed in Africa, largely owing to resource constraints. This study sought to establish the nature of stroke cases (types) seen in Kenya’s two leading referral hospitals, Kenyatta National Hospital (KNH) and Moi Teaching and Referral Hospital (MTRH). It also sought to establish the prevalence of known cerebrovascular risk factors for the stroke subtypes, and ascertain the proportion of first-ever-in-a-lifetime stroke patients and recurrent cases in an effort to provide baseline data for the development of a stroke registry in Kenya. This article highlights the key findings and opportunities for advancing neuroscience in Kenya and sub-Saharan Africa.

## Methods

The study was carried out at KNH located in Nairobi, the capital city of Kenya, and MTRH located in Eldoret, western Kenya. KNH is the leading public tertiary hospital in Kenya with a bed capacity of 1 800, whose occupancy can go up by 300%. The hospital is frequented by patients from all over Kenya, but mostly from the urban areas (Nairobi and its surroundings). MTRH is the second largest public tertiary hospital with an 850-bed capacity that serves patients mainly from the western and Rift Valley regions in Kenya, which are predominantly rural.

Permission to conduct this study was obtained from the Kenya Medical Research Institute (KEMRI) Scientific and Ethics Review Unit (SSC No. 2851), the MTRH institutional research and ethics committee (IREC/2014/213 approval number 0001279) and the Kenyatta National Hospital/University of Nairobi ethics review committee (study registration number MED/029/2015). Informed consent was obtained from each subject or guardian prior to participation in the study. Additional protection for vulnerable populations was put in place to ensure protection of patients’ rights and welfare.

This was a prospective cohort study in which patients were recruited upon admission, and general information on demographics, stroke events and case management was collected using the WHO Stroke STEPS instrument.^7^ Follow up involved assessing clinical outcome at day 10 and 28, and month 3, 6 and 9 using the Modified Rankin Scale,^8^ and gathering information on patient management after discharge.

The study population included all stroke patients diagnosed and/or attended to in KNH and MTRH for the 12-month period between February 2015 and January 2016. The inclusion criteria were confirmed cases of stroke [based on computerised tomography (CT) scan and/or magnetic resonance imaging] treated in out-patient clinics or admitted in hospitals, and in-hospital patients who suffered stroke while on treatment for other illnesses.

The sample size required for the study was based on an unknown proportion of most prevalent stroke type (therefore 50% assumed), a desired precision for the indicator of 5%, and 95% confidence level. Fisher’s formula^9^ for estimating the minimum sample size for descriptive studies was used, giving a minimum sample of 385. The current sample size comprised all recruited stroke patients from the two hospitals in the one-year period.

The combination approach using hot (i.e. active, on-going recruitment) and cold (i.e. retrospective record review) casefinding methods was used to ensure complete identification of stroke cases. Patients were identified from the hospital registry, out-patient clinics, in-patient wards, emergency room and intensive care units. Referrals to specialist physicians or neurologists, physiotherapists, speech or occupational therapists were also monitored to avoid missing any cases. Discharge records and death certificates were scrutinised for stroke diagnosis. Care was taken to avoid duplicate reporting of cases by counterchecking with the hospital electronic database.

The study utilised a modified WHO STEP-wise approach to stroke surveillance tool designed to collect in a standardised manner, basic epidemiological data on incidence, major risk factors, morbidity and mortality trends, and intervention strategies in recent (acute) stroke.^10^ The tool was administered through face-to-face interviews with patients and/or the contact person(s). Follow-up interviews were done where possible physically and/or by telephone.

Data were collected on all aspects of stroke, including information on patient demographic details (gender, age and residence), date of stroke diagnosis, stroke subtype (ischaemic or haemorrhagic), single or multiple strokes, and history of cigarette smoking prior to the current stroke. Information related to care, such as whether or not a CT scan was done was also collected. Regular follow up visits were done every three months to ascertain the patient status after discharge.

## Statistical analysis

Data were analysed using SPSS version 20, with p < 0.05 considered statistically significant. Results are expressed as means ± SD or as proportions (%). For categorical variables, the chi-squared test and Fisher’s exact probability were used. Linear associations were calculated using the Spearman correlation coefficient.

## Results

A total of 691 patients with confirmed stroke [KNH 406 (males: 40.9%; females: 59.1%); MTRH 285 (males: 44.6%; females: 55.4%)] were recruited; 293 (42.4%) were males and 398 (57.6%) were females, giving a male:female ratio of 1:1.4. The median age was 60 years [interquartile range (IQR): 45–73 years], with a minimum of 18 and maximum of 115 years.

Overall, ischaemic stroke accounted for 55.6% of the stroke cases. The occurrence of ischaemic stroke in MTRH was significantly higher (KNH, 50.9%; MTRH 62.1%) than haemorrhagic stroke (KNH 49.1%; MTRH 31.9%) (p = 0.002).

**Fig. 1:**
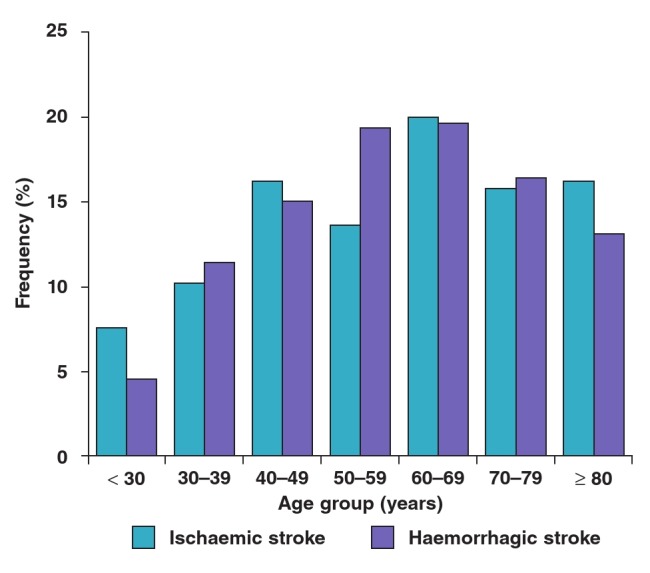
Distribution of stroke type by age.

There were more women diagnosed with stroke than men (57.5 vs 42.5%, respectively). Ischaemic stroke was more prevalent in females (males: 41.1%; females: 58.9%) compared to haemorrhagic stroke (males: 44.4%; females: 55.6%). The occurrence of stroke increased with increasing age. The distribution of stroke across the age groups ≤ 30, 30–39, 40–49, 50–59, 60–69, 70–79 and ≥ 80 years was 6.3, 10.8, 15.8, 16.2, 19.9, 16.1 and 14.9%, respectively. Up to 82.9% of all the stroke patients were 40 years and older. Cases of haemorrhagic stroke were highest in those aged 50–69 years, while ischaemic stroke peaked in the 60–69 age group. Fig. 1 shows the distribution of stroke type by age group.

Among the 691 patients recruited, 106 (15.4%) had suffered a recurrent stroke, among whom 55.6% were women. A significant association was observed between increasing age and recurrent stroke (p = 0.05).

Mortality rate at day 10 and 28 in KNH was 18 and 8.4%, respectively, whereas in MTRH it was 15.5 and 10.3%, respectively. Mortality rate at month 3, 6 and 9 was 10.6, 5.4 and 1.6% at KNH and 12, 8.2 and 8.8% at MTRH, respectively. Table 1 shows the distribution of mortality rate at day 10 and 28 and month 3, 6 and 9 of follow up by gender, health facility and stroke type.

More deaths occurred among haemorrhagic stroke patients by day 10 (20.3%) and day 28 (9.9%) compared to the ischaemic cases (14.6 and 8.9%, respectively), although this was not statistically significant. Thereafter, mortality rate was significantly higher in ischaemic stroke at month 3 (p = 0.027) and month 6 (p = 0.006), and more so in MTRH at month 3 (p = 0.010) and month 6 (p = 0.011).

**Table 1:** Distribution of stroke mortality rates at day 10 and 28, and month 3, 6 and 9 by gender, health facility and stroke type

	*Day 10*		*Day 28*		*Month 3*		*Month 6*		*Month 9*	
	*Alive (%)*	*Dead (%)*	*Alive (%)*	*Dead (%)*	*Alive (%)*	*Dead (%)*	*Alive (%)*	*Dead (%)*	*Alive (%)*	*Dead (%)*
Number	549	113	475	49	340	44	206	15	90	4
Male	84.2	14.7	87.8	10.9	85.6	11.4	89.1	5.9	92.7	4.9
Female	81.3	18.7	91.6	8.1	87.9	11.2	92.1	7.1	96.3	3.7
KNH	82.0	18.0	91.6	8.4	89.4	10.6	94.6	5.4	98.4	1.6
MTRH	83.4	15.5	87.9	10.3	84.0	12.0	85.7	8.2	88.2	8.8
Ischaemic stroke	84.6	14.6	90.1	8.9	83.3	14.0	85.5	9.9	89.8	8.2
Haemorrhagic stroke	79.7	20.3	89.6	9.9	92.5	6.9	97.9	2.1	100	0

The most common risk factors were hypertension [77.3% (males: 75.7%; females: 78.5%)], cigarette smoking [16.1% (males: 26.6%; females: 8.3%) p < 0.001], diabetes [14.9% (males: 15.7%; females: 14.4%)], and hypercholesterolaemia [2.8% (males: 4.1%; females: 1.8%) p < 0.05]. Other factors associated with stroke were history of previous migraine [32.8% (males: 28.3%; females: 36.0%) p < 0.05], HIV infection [8% (males: 7.2%; females: 8.6%)], use of oral contraceptives [3.9% (females: 6.8%)] and cocaine use [0.7% (males: 0.7%; females: 0.8%)]. Table 2 shows the distribution of risk factors for stroke by gender and age group.

**Table 2:** Distribution of risk factors by gender and age group

*Age groups*														
	*< 30 years*		*30–39 years*		*40–49 years*		*50–59 years*		*60–69 years*		*70–79 years*		*≥ 80 years*	
Risk factors	*Men n (%)*	*Women n (%)*	*Men n (%)*	*Women n (%)*	*Men n (%)*	*Women n (%)*	*Men n (%)*	*Women n (%)*	*Men n (%)*	*Women n (%)*	*Men n (%)*	*Women n (%*	*Men n (%)*	*Women n (%)*
Current tobacco use+	1 (9.1)	0 (0)	6 (19.4)	2 (4.7)	12 (27.9)	2 (3)	17 (33.3)	2 (3.2)	18 (27.7)	4 (5.6)	11 (20)	9 (16.4)	13 (35.1)	14 (21.2)
Diabetes mellitus*	1 (9.1)	0 (0)	1 (3.2)	4 (9.3)	2 (4.7)	8 (11.9)	3 (5.9)	4 (6.6)	19 (29.2)	21 (29.2)	14 (25.5)	9 (16.4)	6 (16.2)	11 (16.7)
Hypercholesterolaemia†	0 (0)	1 (3.1)	2 (6.5)	0 (0)	1 (2.3)	0 (0)	2 (3.9)	2 (3.2)	3 (4.6)	1 (1.4)	3 (5.5)	2 (3.6)	1 (2.7)	1 (1.5)
Hypertension‡	5 (45.5)	15 (46.9)	15 (48.4)	25 (59.5)	29 (69)	46 (69.7)	42 (82.4)	49 (79)	57 (87.7)	68 (94.4)	47 (85.5)	51 (92.7)	26 (70.3)	56 (84.8)
Oral contraceptives	0 (0)	6 (18.8)	0 (0)	12 (27.9)	0 (0)	8 (11.9)	0 (0)	0 (0)	0 (0)	1 (1.4)	0 (0)	0 (0)	0 (0)	0 (0)
Previous migraine	4 (36.4)	18 (56.3)	14 (45.2)	19 (44.2)	12 (27.9)	30 (44.8)	16 (31.4)	20 (32.3) 20 (32.3)	20 (30.8)	27 (37.5)	9 (16.4)	14 (25.5)	8 (21.6)	15 (22.7)
HIV infection	0 (0)	7 (21.9)	3 (9.7)	7 (16.3)	9 (20.9)	12 (17.9)	5 (9.8)	5 (8.1)	3 (4.6)	2 (2.8)	1 (1.8)	1 (1.8)	0 (0)	0 (0)
Cocaine use	0 (0)	0 (0)	1 (3.2)	1 (2.3)	0 (0)	1 (1.5)	1 (2)	0 (0)	0 (0)	1 (1.4)	0 (0)	0 (0)	0 (0)	0 (0)

+Cigarette smoking within the last five years. *Elevated fasting glucose level > 5.6 mmol/l or treatment of previously diagnosed diabetes. †High-density lipoprotein cholesterol < 1.3 mmol/l; triglycerides > 2.2 mmol/l; total cholesterol > 6.2 mmol/l; low-density lipoprotein cholesterol > 4.1 mmol/l, or specific treatment for this abnormality.‡Elevated blood pressure 140/90 mmHg or treatment of previously diagnosed hypertension.

A test of conditional independence (Cochran’s Mantel– Haenszel chi-squared) gave χ2 1 = 0.314; p = 0.575, indicating that ischaemic stroke was conditionally independent of gender after adjusting for age. No significant association was observed therefore between ischaemic stroke and gender across the various age groups.

## Discussion

There is scanty information on the extent and nature of stroke in Kenyan public hospitals. This study was set to determine the clinico-epidemiological profile of stroke in Kenya’s leading public health tertiary institutions.

Higher incidence of ischaemic stroke was observed, with hypertension, tobacco use and diabetes as the most common vascular risk factors. These findings agree with those previously reported from Nairobi and Aga Khan private hospitals, where ischaemic stroke was the most common stroke sub-type, and hypertension and diabetes were the leading risk factors.^11^,^12^ An important observation in this study was the distribution of stroke and the associated risk factors in both rural (MTRH) and urban (KNH) regions. This signifies a general shift in lifestyle and demographics, which often accompanies economies in transition, and it is perhaps best substantiated by cigarette smoking as the second most-common risk factor.

Advancing age is the most important predictor of cardiovascular morbidity and mortality. The increased stroke cases observed with increasing age in this study attest to that.^13^ The stroke burden was higher in the 40–79-year age bracket, which represents middle-aged adults, whom as has been stated before, contribute to the 78% stroke burden in low- and middleincome countries.^14^ It has also been shown that high and increasing rates of stroke affect people at much younger ages in sub-Saharan Africa, resulting in greater numbers of years of potential life lost.^15^,^16^ Hence, aggressive efforts in improving cardiovascular health, promoting healthy aging, preventing cardiovascular risk factors and fast-tracking proven intervention strategies are necessary to halt and reverse the CVD burden.^13^,^17^

The post-stroke mortality rate in the current study was higher than the average national estimate of 12% for CVD deaths in hospitals, suggesting poor outcomes in post-stroke events. Similar high fatalities have been observed elsewhere in Africa, with high blood pressure predicting fatality in the short term, particularly with haemorrhagic stroke.^18^ In-patient stroke mortality rate of 19.3% has also been reported in the Congo, 33.3% in Tanzania, 43.2% in Ghana and 23.2% in Cameroon by day 30.18-21 Monthly stroke mortality rates in South Africa are similarly high, with 23% mortality rate reported at month 6.^22^,^23^

Therefore continuous monitoring of stroke incidence, outcomes and determinants should be enhanced to provide the much-needed information for guiding health service provision and allocation of resources. More work is required to assess the impact of actual care patterns on stroke prognosis over time, while prioritising the reduction of haemorrhagic stroke in Kenya and sub-Saharan Africa as a whole.^24^,^25^

Hypertension is the single most important risk factor and contributor to disability and premature death. In our study, the burden of hypertension was equally distributed across gender. This confirms previous findings from sub-Saharan Africa that show hypertension as the most powerful predictor of stroke. The contribution of untreated hypertension to stroke burden has been demonstrated in Ethiopia and Tanzania,^26^,^27^ and reiterates the importance of understanding the primary drivers for effective prevention.^15^,^28^,^29^

Treatment of hypertension can reduce the risk of stroke by more than 40%. There is a need therefore to develop comprehensive risk-reduction strategies to mitigate the social and economic burden of stroke. Renewed emphasis on prevention and control of high blood pressure is necessary.^16^,^30^

Non-communicable diseases are beginning to feature on the public health agenda in developing countries.^31^,^32^ However, despite CVD being the second leading cause of morbidity and mortality in Kenya, its prevention and mitigation of risk factors are yet to receive the warranted attention necessary to protect and improve public health. There is a need to build scientific evidence that will assist in health planning, advocacy and policy making. The Kenyan county governments should deliberately invest in capacity building and harnessing of resources for CVD research and service provision. Supporting the development and sustenance of CVD surveillance systems will enhance knowledge generation and utilisation of evidence in fast-tracking prevention and control measures.

## Conclusions

Ischaemic stroke was the most prevalent stroke at 55.6%. Hypertension was the commonest risk factor, followed by smoking and diabetes, and the overall mortality rate was higher than that estimated by the WHO. Variation in stroke occurrence was observed based on gender and increasing age. There is a need to implement and/or scale up proven interventions geared towards preventing and controlling stroke and the associated risk factors, while being cognisant of the socio-demographic and cultural changes accompanying economies in transition. In addition, raising the population’s awareness of lifestyle factors likely to predispose them to stroke, and investing in care, management and surveillance systems may, with time, reduce the number of cases of stroke, initial stroke severity and improve public health.
